# BCAT1 promotes acetylation of ACLY through fatty acid metabolism pathway promoting the occurrence and development of endometriosis

**DOI:** 10.3389/fgene.2026.1818692

**Published:** 2026-10-20

**Authors:** Yanan Xu, Yanping Shen, Yingyan Yan, Liang Chen

**Affiliations:** 1 Department of Scientific Research, The Affiliated Women and Children’s Hospital of Ningbo University, Ningbo, Zhejiang Province, China; 2 Department of Anesthesiology, The Affiliated Women and Children’s Hospital of Ningbo University, Ningbo, Zhejiang Province, China; 3 GCP Center, The Affiliated Women and Children’s Hospital of Ningbo University, Ningbo, Zhejiang Province, China; 4 Department of Gynecology, The Affiliated Women and Children’s Hospital of Ningbo University, Ningbo, Zhejiang Province, China

**Keywords:** ACLY, BCAT1, endometriosis, fatty acid metabolism, histone acetylation

## Abstract

**Objective:**

Currently, surgical intervention is the primary treatment for endometriosis (EM), but recurrence is common. This study aims to identify novel potential therapeutic targets for effective management of EM by investigating the role of branched chain amino acid transaminase 1 (BCAT1).

**Methods:**

We analyzed BCAT1 expression in EM tissues using quantitative reverse transcription polymerase chain reaction (qRT-PCR) and Western blot analysis. The functional impact of BCAT1 on ectopic endometrial stromal cells (EESCs) was evaluated using CCK-8, colony formation, wound healing, and Transwell assays. To study BCAT1’s role in fatty acid metabolism, we conducted transcriptomic sequencing, oxygen consumption rate (OCR) assays, lipid droplet assays, and measured cholesterol and triglyceride levels. The interaction between BCAT1 and ATP-citrate lyase (ACLY) was further analyzed through Western blotting and BCAT1 modulation experiments.

**Results:**

BCAT1 expression was significantly elevated in EM tissues. Functional assays demonstrated that BCAT1 promotes the proliferation, migration, and invasion of EESCs *in vitro*. BCAT1 also positively regulates fatty acid metabolism, which enhances ACLY expression and contributes to cell proliferation. Mechanistically, BCAT1 influences ACLY expression through epigenetic regulation, specifically via histone acetylation.

**Conclusion:**

BCAT1 plays a crucial role in regulating EESC functions by modulating fatty acid metabolism and ACLY expression. These findings provide a theoretical basis for EM treatment.

## 1 Introduction

Endometriosis (EM) is a prevalent inflammatory disorder characterized by the presence of endometrial-like tissue outside the uterus (Zondervan et al., 2018). The diagnosis of EM is primarily established through visual confirmation via surgical procedures, most commonly laparoscopic surgery (Zondervan et al., 2020). A population-based estimate from 2017 indicates that EM affects approximately 10% of women of reproductive age (Shafrir et al., 2018). Symptoms of EM include chronic pelvic pain, menstrual discomfort, cyclical gastrointestinal symptoms, and infertility (Carpinello et al., 2000), all of which substantially impact individuals’ quality of life (Kuznetsov et al., 2017). At present, conservative surgery remains a treatment option for EM; however, it does not prevent disease recurrence (Saunders and Horne, 2021). Due to the hormone-dependent nature of EM, hormonal therapy is another common treatment modality (Zakhari et al., 2021; Lyons et al., 2006). Despite its prevalence, this therapy poses significant side effects and is not a viable long-term option for women wishing to conceive (Park et al., 2024). Consequently, it is critical to explore new molecular mechanisms and identify potential prognostic markers and potential therapeutic targets for the effective management of EM.

Branched-chain amino acids (BCAAs) play a crucial role in disease progression, with branched-chain amino acid transaminase 1 (BCAT1) being one of the key enzymes that catalyze the first step of BCAAs degradation (Ananieva, 2015; Tönjes et al., 2013). BCAT1 is a cytosolic enzyme that can convert leucine, isoleucine, and valine into their corresponding branched-chain α-keto acids (Yuan et al., 2024). BCAT1 has become a focal point of research in the progression of various diseases, as it is highly expressed in multiple disease tissues and promotes cell proliferation (Panosyan et al., 2017; Ouyang et al., 2022; Chen et al., 2025). For instance, overexpressing BCAT1 can enhance the proliferation, migration, and invasion of nasopharyngeal carcinoma (NPC) cells (Zhou et al., 2013). BCAT1 is an important target for treating muscle atrophy. Knocking down BCAT1 effectively inhibits muscle cell proliferation and induces a significant increase in reactive oxygen species (ROS) production (Ouyang et al., 2022). Additionally, the study shows that knocking down BCAT1 can reduce the growth rate of cardiomyocytes and promote ferroptosis to enhance Keap1 expression, providing new insights for the treatment of myocardial injury (Fu et al., 2022). However, the role of BCAT1 in EM has not been reported and requires further investigation to elucidate its mechanisms.

Fatty acids, as the main components of lipids, play an important role in regulating the structure and dynamics of biological membranes, energy storage, and signal transduction (Rohrig and Schulze, 2016). The reprogramming of fatty acid metabolism promotes tumorigenesis, disease progression, and treatment resistance by enhancing the synthesis, storage, and breakdown of lipids (Hoy et al., 2021). This metabolism is closely related to cancer signaling pathways and is a key factor in promoting disease progression (Golay et al., 1986). Generally, cells utilize lipid metabolism to obtain energy, generate components for biological membranes, and support cell proliferation and metastasis (Principe et al., 2024). However, fatty acid metabolism in EM has been rarely studied, and the specific molecular mechanisms remain unclear. Therefore, deepening the understanding of fatty acid metabolism in EM cells is crucial for developing more effective and targeted therapeutic interventions.

ATP-citrate lyase (ACLY) is a key enzyme that plays a crucial role in metabolism by converting citrate and coenzyme A into acetyl-coenzyme A (acetyl-CoA) and oxaloacetate. Acetyl-CoA serves as an essential component for *de novo* lipogenesis (DNL) and the acetylation modification of target proteins (Icard et al., 2020). ACLY also connects energy metabolism with lipid synthesis, thereby promoting cell growth (Ward and Thompson, 2012). Additionally, ACLY is implicated in cancer; for instance, the combined application of ACLY inhibitors and PD-L1 antibodies significantly suppressed chemically induced hepatocellular carcinoma (HCC) (Gu et al., 2021). Downregulation of ACLY via small interfering RNA can inhibit the proliferation and migration of renal cancer cells while promoting their apoptosis (Teng et al., 2018). In lung cancer, ACLY inhibition results in growth stagnation both *in vitro* and *in vivo* (Migita et al., 2008). Studies have identified ACLY as a potential therapeutic target for cancer, leading to the development of several ACLY inhibitors (Hu et al., 2017). Notably, the ACLY inhibitor bempedoic acid has been approved by the FDA for the treatment of hypercholesterolemia, demonstrating evidence of reducing adverse cardiac events (Krishna Mohan et al., 2023).

This study initially analyzes the expression of the BCAT1 protein in normal uterine tissue compared to EM tissue. It subsequently investigates the biological behaviors of BCAT1 in EM cells through experiments such as CCK-8, wound healing, and Transwell invasion assays. Bioinformatics analysis predicts BCAT1-related signaling pathways, which are then verified through Western blotting. In summary, we examined the expression, function, and potential mechanisms of BCAT1 in EM, providing a theoretical basis for EM treatment.

## 2 Materials and methods

### 2.1 Tissue samples

Tissue samples were collected from 30 patients with endometriosis and 30 normal individuals at The Affiliated Women and Children’s Hospital of Ningbo University. Ectopic endometriotic lesions and paired eutopic endometrial tissues (from the uterine cavity) were obtained from each patient with endometriosis, while normal endometrial tissues were collected from the control individuals. Among the collected samples, 6 paired cases were used for Western blot analysis, and 3 paired cases were used for primary cell isolation. All participants provided written informed consent. This study received approval from the Ethics Committee of The Affiliated Women and Children’s Hospital of Ningbo University (Ethics number: EC2022-028) and conformed to the Declaration of Helsinki. All endometriosis cases were diagnosed through laparoscopic surgery combined with pathologic examination.

### 2.2 Cell isolation and cell culture

Primary endometrial stromal cells were isolated from ectopic endometrial tissues and normal control endometrial tissues obtained from 3 independent patients (n = 3 for ectopic, n = 3 for normal). The isolated cells were designated as primary ectopic endometrial stromal cells (EESCs) and normal endometrial stromal cells (NESCs), respectively. Cells from each independent patient-derived preparation were cultured separately in DMEM supplemented with 10% fetal bovine serum (FBS) at 37 °C in a 5% CO_2_ incubator. All experiments were performed with at least three independent biological replicates (i.e., cells derived from three different patients per condition), and each biological replicate was tested in triplicate technical repeats (or as appropriate). The passage number of cells used for experiments was kept between 2 and 4.

### 2.3 Cell transfection

To achieve BCAT1 and ACLY knockdown in endometrial stromal cells, we used specific small interfering RNAs (siRNAs) targeting BCAT1 (siBCAT1) and ACLY (siACLY), respectively. The siRNA sequences are provided in Supplementary Table S1. Transfections were performed using Lipofectamine 2000 reagent (Invitrogen) according to the manufacturer’s instructions. Briefly, cells were seeded in 6-well plates and transfected with 100 nM of each siRNA when they reached 60%–70% confluence. The transfection medium was replaced with complete growth medium 6 h post-transfection, and cells were harvested at 48 h for subsequent assays.

To achieve transient overexpression of BCAT1, endometrial stromal cells were transfected with pcDNA3.1-BCAT1 or pcDNA3.1 empty vector (negative control) using Lipofectamine 2000 reagent. Cells were seeded in 6-well plates at 4 × 10^5^ cells/well 1 day before transfection. At 70% confluence, 2 µg of plasmid DNA and 4 µL of Lipofectamine 2000 were each diluted in 100 µL of Opti-MEM™ I Reduced Serum Medium (Gibco), combined, and incubated at room temperature for 5 min. The DNA–liposome complexes were added dropwise to the cells and incubated at 37 °C in 5% CO_2_. After 6 h, the medium was replaced with fresh complete medium. Cells were collected at 48 h post-transfection for Western blotting and qRT-PCR analysis.

### 2.4 qRT-PCR

Total RNA was extracted from EESCs using TRIzol reagent (TransGen Biotech). Subsequently, complementary DNA (cDNA) was synthesized using a reverse transcription kit (Vazyme). Quantitative reverse transcription polymerase chain reaction (qRT-PCR) (qRT-PCR) was performed with SYBR Green (Vazyme), using GAPDH as the endogenous reference on a CFX96 Real-Time PCR Detection System. The primers utilized in this study are listed in Supplementary Table S2.

### 2.5 Western blot assay

Cellular proteins were lysed using RIPA lysis buffer (Beyotime Biotechnology). Equal amounts of protein samples were separated on a 10% Tris-Bis gel and subsequently transferred onto a PVDF membrane. The membrane was blocked with 5% skim milk in TBST for 1 h at room temperature, washed three times with TBST, and then incubated overnight at 4 °C with primary antibodies (details are provided in Supplementary Table S3). After washing three times with TBST, the membrane was incubated with horseradish peroxidase-conjugated secondary antibodies for 1 h at room temperature. Finally, the protein bands were visualized using a chemiluminescence gel imaging system.

### 2.6 Cell counting kit-8 (CCK-8) assay

The proliferative capacity of EESCs was assessed using the CCK-8 assay. Cells (4 × 10^3^ cells/well) were cultured in a 96-well plate (NEST) for 0, 24, 48, 72, and 96 h. After stable cell adhesion, CCK-8 solution (Beyotime Biotechnology) was added to the wells and incubated for 2.5 h at 37 °C. The results were obtained using a microplate reader, measuring absorbance at 450 nm.

### 2.7 Colony formation assay

The cells (1000/well) were cultured in the 6-well plates (NEST). After 14 days, colonies were washed three times with 1 × PBS, fixed with 4% paraformaldehyde (Solarbio) for 25 min at room temperature, stained with 0.5% crystal violet (Solarbio) for 40 min, and then counted manually.

### 2.8 Wound healing assay

Cells were cultured in 6-well plates to 80%–90% confluence. Before wounding, cells were pretreated with mitomycin C (1 μg/mL) for 2 h to suppress proliferation, followed by serum starvation for 12 h to synchronize the cell cycle. A straight scratch was then made across each well using a sterile 200 μL pipette tip. After washing twice with PBS to remove detached cells, the cultures were switched to serum-free medium containing mitomycin C (1 μg/mL). Phase-contrast images were taken immediately (0 h) and after 24 h of incubation. The wound width was measured at multiple fixed points using Image-Pro software, and the percentage of wound closure was calculated as [(width_0_ − width_24_)/width_0_] × 100%.

### 2.9 Transwell invasion assay

Matrigel (BD Biosciences) was pre-coated onto the upper surface of the Transwell inserts. Cells were collected, resuspended in serum-free medium, and seeded into the upper chambers. The lower chambers were filled with medium containing 15% fetal bovine serum (Gibco) as a chemoattractant. After 24 h of incubation, cells remaining on the upper surface of the membrane were gently removed with a cotton swab. The invaded cells on the lower surface were fixed, stained, and counted under a microscope. The number of invaded cells was quantified by counting five random fields per insert. For the migration assay, the procedure was identical except that the inserts were not coated with Matrigel. Both assays were performed with three independent biological replicates (cells from three different patients), with three inserts per condition and five random fields counted per insert.

### 2.10 Lipid droplet staining assay

The cells were fixed with 4% paraformaldehyde for 15 min at room temperature. And then they were stained with BODIPY 493/503 (Invitrogen) for 30 min to stain the lipid droplets, subsequently treated with DAPI for 5 min, and washed with PBS gently. The results were visualized using a confocal laser scanning microscope (Leica).

### 2.11 The OCR detection

The OCR was detected by an XFe24 extracellular flux analyzer (Seahorse Bioscience, Billerica, MA). Before the test, the 20,000 cells were cultured in an XFe24 cell culture microplate in growth medium. It conducted follow-up experiments according to the instructions.

### 2.12 Cholesterol and triglycerides content assays

According to the manufacturer’s instructions, the cholesterol and triglycerides content of the cells assay was performed with a commercial kit.

### 2.13 Chromatin immunoprecipitation followed by quantitative PCR (ChIP-qPCR)

ChIP was performed using the ChIP Assay Kit (P2078, Beyotime Biotechnology, Shanghai, China) according to the manufacturer’s protocol. Cells were cross-linked with 1% formaldehyde at 37 °C for 10 min, and the reaction was quenched with Glycine Solution for 5 min at room temperature. Cells were washed twice with ice-cold PBS containing 1 mM PMSF and collected by centrifugation. Cell pellets were lysed in SDS Lysis Buffer containing 1 mM PMSF and incubated on ice for 10 min. Chromatin was sheared by sonication to obtain DNA fragments of 200–1,000 bp. After centrifugation, the supernatant was diluted with ChIP Dilution Buffer containing 1 mM PMSF. A portion of the diluted chromatin was saved as input control. The remaining chromatin was pre-cleared with Protein A + G Agarose/Salmon Sperm DNA at 4 °C for 1 h. After centrifugation, the supernatant was incubated overnight at 4 °C with a specific antibody against the target protein (4 µg) or with normal IgG (4 µg) as a negative control. Immune complexes were captured by adding Protein A + G Agarose/Salmon Sperm DNA and incubating at 4 °C for 2 h. The agarose beads were washed sequentially with Low Salt Immune Complex Wash Buffer, High Salt Immune Complex Wash Buffer, LiCl Immune Complex Wash Buffer, and twice with TE Buffer. Bound chromatin was eluted in elution buffer. Cross-linking was reversed by incubation with proteinase K at 65 °C overnight. DNA was purified using a commercial ChIP DNA purification kit and eluted in nuclease-free water. qPCR data were normalized to input and expressed as fold enrichment over IgG control, as described separately.

### 2.14 RNA-sequencing

Total RNA was extracted from EESCs transfected with si-NC or si-BCAT1 using TRIzol reagent (Invitrogen) according to the manufacturer’s instructions. RNA integrity and concentration were assessed using an Agilent 2100 Bioanalyzer (RNA Integrity Number ≥7.0). Three independent biological replicates were prepared per group. Library construction was performed using the NEBNext Ultra II Directional RNA Library Prep Kit for Illumina, and sequencing was carried out on an Illumina NovaSeq 6,000 platform with paired-end 150 bp reads. Raw reads were filtered using fastp (v0.20.0) to remove adapters and low-quality bases. Clean reads were aligned to the human reference genome (GRCh38) using HISAT2 (v2.2.1). Gene expression levels were quantified as fragments per kilobase of transcript per million mapped reads (FPKM) using StringTie (v2.1.5). Differentially expressed genes (DEGs) were identified using DESeq2 (v1.34.0) with the thresholds of |log_2_ fold change| ≥ 1 and adjusted P value <0.05 (Benjamini–Hochberg correction). KEGG pathway enrichment analysis of DEGs was performed using clusterProfiler (v4.2.2).

### 2.15 Statistical measurement

All statistical analyses were performed using GraphPad Prism 8.0. Data are presented as mean ± standard deviation (SD) and are representative of at least three independent biological replicates, each performed in triplicate technical replicates. Normality of data distribution was assessed using the Shapiro-Wilk test. For comparisons between two groups, a two-tailed unpaired Student’s t-test was used. For comparisons among three or more groups, one-way analysis of variance (ANOVA) followed by Tukey’s *post hoc* test for multiple comparisons was applied. For experiments involving two independent variables, two-way ANOVA followed by Sidak’s *post hoc* test was used. A P value <0.05 was considered statistically significant. Significance levels are denoted as follows: ns, not significant; *P < 0.05; P < 0.01; *P < 0.001. Exact P values are reported in figure legends where applicable.

## 3 Results

### 3.1 BCAT1 is highly expressed in EM

To investigate the changes of BCAT1 expression in EM, we assessed the BCAT1 expression using qRT-PCR and Western blot in the ectopic (EC) group, eutopic (EU) group, and normal control (NM) group. We confirmed a significant increase in BCAT1 expression in EM tissues (Figures 1A,B). To further validate our analysis, we analyzed the BCAT1 expression by using the GSE120103 database. We found that the BCAT1 expression was significantly increased in EM (Figure 1C). Next, we evaluated BCAT1 expression in primary normal (NESC) and ectopic (EESC) endometrial stromal cells. The results indicated BCAT1 expression was significantly increased in EESCs (Figure 1D). Finally, ROC curve analysis revealed that BCAT1 expression exhibited high sensitivity and discriminatory capacity in distinguishing EM tissues from normal controls (Figure 1E). In summary, our study found BCAT1 is highly expressed of in EM.

**FIGURE 1:**
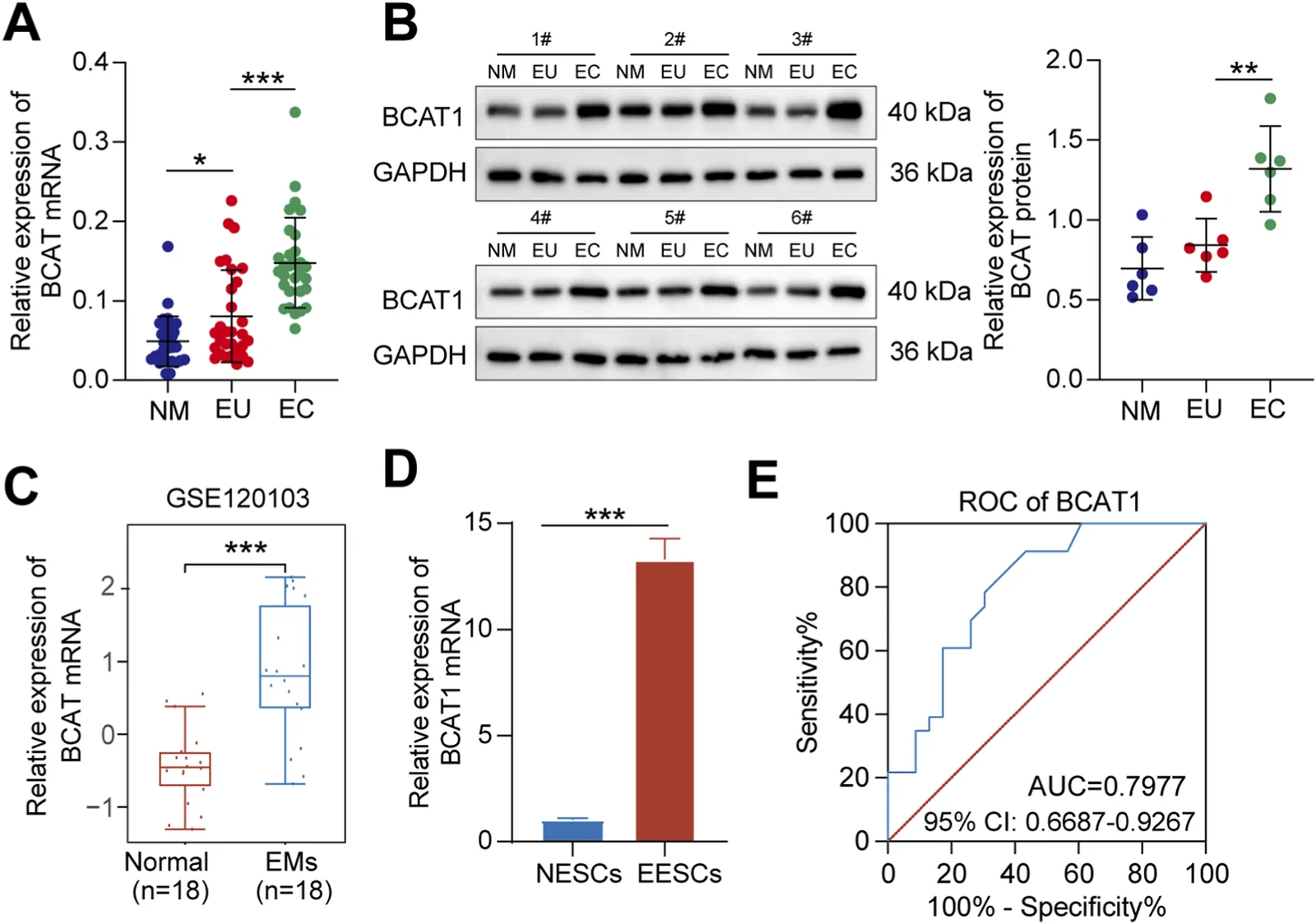
BCAT1 is highly expressed in EM. **(A)** qRT-PCR analysis of BCAT1 mRNA expression in ectopic (EC), eutopic (EU), and normal (NM) endometrial tissues. Each group comprised n = 30 independent patient tissue samples (biological replicates); each sample was analysed in three technical PCR replicates and the results were averaged. **(B)** Western blot analysis of BCAT1 protein expression in each group. Each group comprised n = 6 independent patient tissue samples (biological replicates); a representative blot is shown, and band intensities were quantified across all six independent samples. **(C)** Validation of BCAT1 expression in the public microarray dataset GSE120103. Each group comprised n = 18 independent samples (biological replicates); no technical replicates were used for this microarray analysis, and each sample was measured once. **(D)** qRT-PCR analysis of BCAT1 expression in primary endometrial stromal cells (endometriosis-derived EESCs and normal NESCs). Each group comprised n = 3 independent patient-derived cell preparations (biological replicates); each preparation was analysed in three technical PCR replicates and the results were averaged. **(E)** ROC curve analysis of the diagnostic value of BCAT1 for endometriosis, based on the 30 ectopic and 30 normal tissue samples described in **(A)** (n = 30 independent biological samples per group); AUC = 0.7977 (95% CI 0.6687–0.9267). Data are presented as mean ± SD. Comparisons among three groups **(A,B)** were performed using one-way ANOVA followed by Tukey’s *post hoc* test; comparisons between two groups **(C,D)** were performed using two-tailed unpaired Student’s t-tests, with the microarray data **(C)** analysed after normalization. ns, not significant; *P < 0.05; **P < 0.01; ***P < 0.001.

### 3.2 BCAT1 enhances the proliferation of EM cells *in vitro*

To further study the specific impact of BCAT1 on EM cell function, we transfected two independent siRNAs into EESCs to knock down BCAT1 and constructed BCAT1 overexpressing cells. First, qRT-PCR validated reduced BCAT1 expression after knockdown and increased expression upon overexpression in EESCs (Figures 2A,B). Next, CCK-8 results demonstrated BCAT1 knockdown inhibited EESC proliferation, while overexpression promoted it (Figure 2C). Furthermore, colony formation assays revealed that BCAT1 knockdown reduced EESC proliferation, whereas BCAT1 overexpression enhanced it (Figure 2D). Importantly, to further demonstrate that the reduction in cell proliferation was indeed due to knocking down BCAT1, we overexpressed BCAT1 in EESCs with BCAT1-knockdown EESCs. CCK-8 and colony formation assays indicated that overexpression of BCAT1 significantly restored the proliferative capacity of BCAT1-knockdown EESCs (Supplementary Figures S1A,B). Overall, these findings suggested that BCAT1 promoted the proliferation of EM cells *in vitro*. Subsequently, to investigate the effect of BCAT1 on the migratory and invasive ability of EESCs, we conducted the wound healing and Transwell assays. The wound healing assay showed that knocking down BCAT1 reduced the migration distance and rate of EESCs, while overexpressing BCAT1 significantly enhanced them (Figure 2E). Additionally, the Transwell assay indicated that knocking down BCAT1 significantly decreased the invasive and migratory capacity of EESCs, whereas overexpressing BCAT1 significantly enhanced them (Figure 2F). Notably, to further illustrate that the reduction in cell migratory capacity was indeed due to knocking down BCAT1, we overexpressed BCAT1 in EESCs with knocking down BCAT1. The wound healing and Transwell assays showed that overexpressing BCAT1 significantly restored the migratory capacity of EESCs with knocking down BCAT1 (Supplementary Figures S1C,D). Interestingly, Western blot analyses revealed that BCAT1 knockdown upregulated E-cadherin and downregulated N-cadherin in EESCs, whereas BCAT1 overexpression exerted the opposite effects (Figure 2G). In summary, our results indicated that BCAT1 promotes the proliferation, migration, and invasion of EESCs *in vitro*.

**FIGURE 2:**
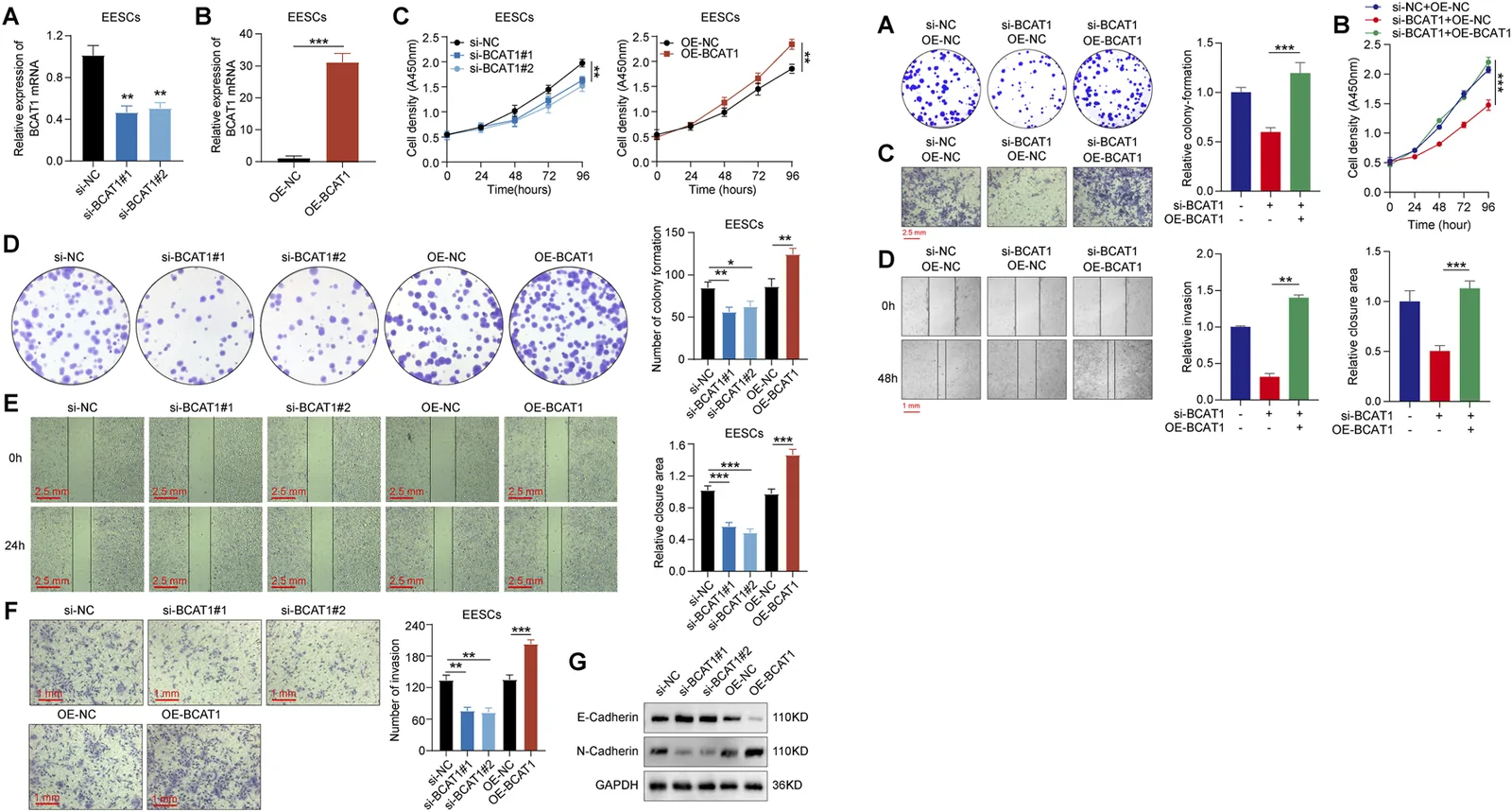
BCAT1 enhances the proliferation of EM cells *in vitro*. **(A,B)** qRT-PCR analysis of BCAT1 mRNA in EESCs after knockdown **(A)** and overexpression **(B)**. Each group comprised n = 3 independent patient-derived cell preparations (biological replicates); each preparation was analysed in three technical PCR replicates and the results were averaged. **(C,D)** CCK-8 **(C)** and colony formation **(D)** assays showed that BCAT1 knockdown reduced, whereas overexpression enhanced, EESC proliferation; each group comprised n = 3 biological replicates, with three replicate wells per time point **(C)** and three replicate wells **(D)**. **(E)** Wound healing assay showed that BCAT1 knockdown reduced, whereas overexpression enhanced, EESC migration; n = 3 biological replicates with three replicate wells and multiple fixed measurement points per well. **(F)** Transwell assay showed that BCAT1 knockdown reduced, whereas overexpression enhanced, EESC invasion and migration; n = 3 biological replicates with three inserts and five random fields counted per insert (invasion with Matrigel, migration without Matrigel). **(G)** Western blotting of EMT markers showed that BCAT1 knockdown decreased N-cadherin and increased E-cadherin expression, with the opposite pattern after overexpression; n = 3 biological replicates with band intensities quantified by densitometry (representative blots shown). Data are presented as mean ± SD. Comparisons between two groups **(A,B,D–G)** were performed using two-tailed unpaired Student’s t-tests; CCK-8 time-course data **(C)** were analysed using two-way ANOVA followed by Sidak’s *post hoc* test. ns, not significant; *P < 0.05; **P < 0.01; ***P < 0.001.

### 3.3 Knocking down BCAT1 reduces lipid accumulation in EM

To further investigate the primary functions and molecular mechanisms of BCAT1, we performed transcriptome sequencing on EESCs from the control group and group with knocking down BCAT1. Volcano plot analysis revealed that 3,025 genes were upregulated, while 4,641 genes were downregulated (Figure 3A). Furthermore, KEGG enrichment analysis revealed significant alterations in the fatty acid biosynthesis pathway in EESCs upon BCAT1 knockdown relative to controls (Figure 3B). Notably, heatmap results showed that after knocking down BCAT1, the fatty acid biosynthsis-related genes expression in EESCs was downregulated to varying degrees, with ACLY showing a significant decrease (Figure 3C). In addition, we found that knocking down BCAT1 resulted in a reduced oxygen consumption rate (OCR) in EESCs. Conversely, overexpressing BCAT1 increased the OCR in EESCs (Figure 3D).

**FIGURE 3:**
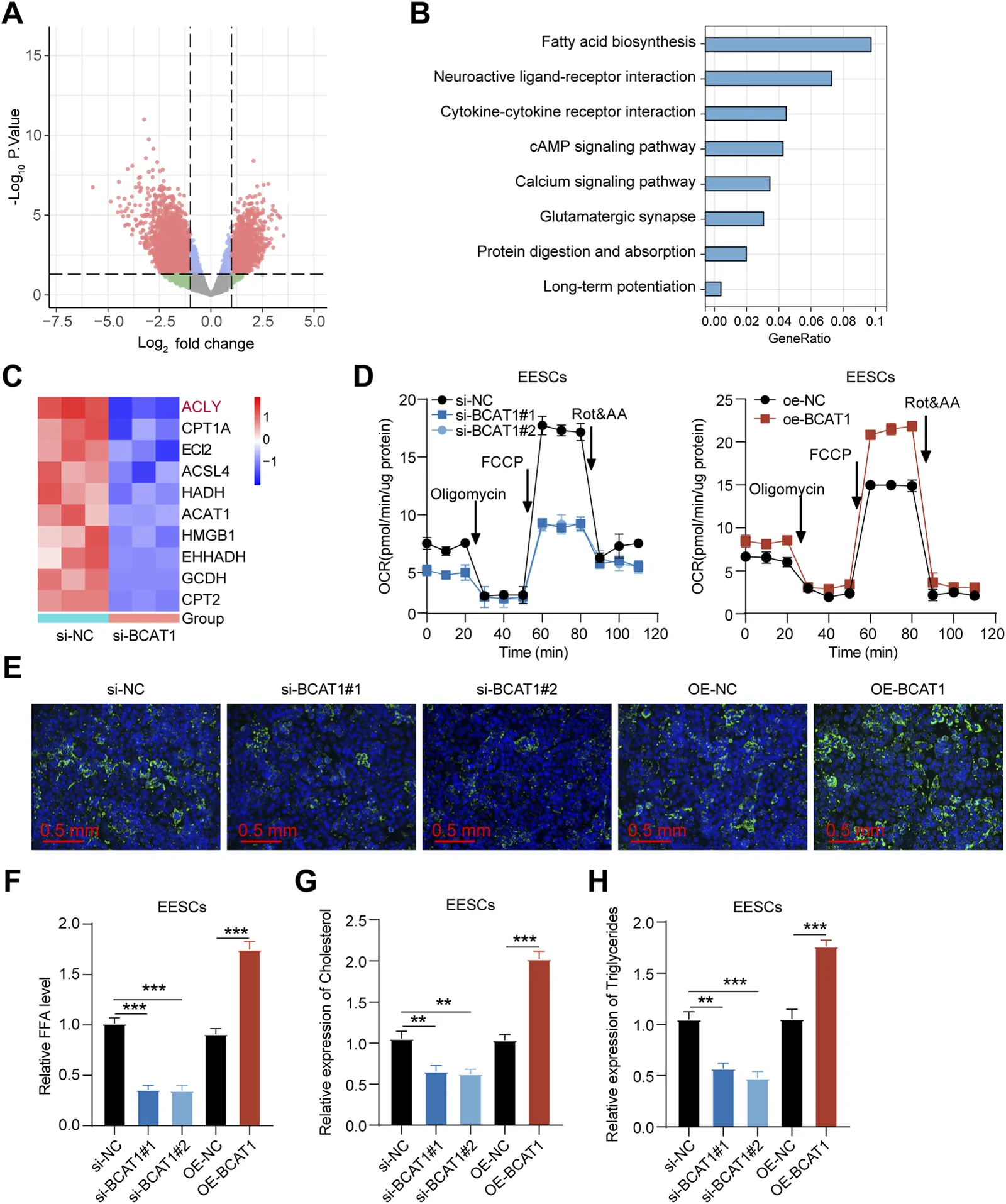
Knocking down BCAT1 reduces lipid accumulation in EM. **(A)** Transcriptomic analysis of BCAT1-knockdown EESCs (si-NC versus si-BCAT1); n = 3 biological replicates per group with one library per replicate (no technical replicates); differentially expressed genes are shown in a volcano plot (|log2 fold change| ≥ 1 and adjusted P < 0.05 by DESeq2). **(B)** KEGG pathway analysis of the differentially expressed genes revealed significant enrichment in fatty acid synthesis (n = 3 biological replicates). **(C)** Clustering heatmap of differentially expressed genes in the fatty acid synthesis pathway showed marked differences in ACLY (n = 3 biological replicates). **(D)** OCR measured in EESCs decreased after BCAT1 knockdown and increased after BCAT1 overexpression; n = 3 biological replicates with three technical wells per condition. **(E,F)** BODIPY staining showed reduced lipid levels after BCAT1 knockdown and increased levels after overexpression; n = 3 biological replicates with three technical repeats per condition. **(G,H)** Total cholesterol **(G)** and triglyceride **(H)** assays showed decreased levels after BCAT1 knockdown and increased levels after overexpression; n = 3 biological replicates with three technical repeats per condition. Data are presented as mean ± SD. DEG identification **(A)** and KEGG enrichment analysis **(B)** were performed using DESeq2 and clusterProfiler, respectively, as described in the Methods; OCR data **(D)** were analysed using two-way ANOVA followed by Sidak’s *post hoc* test; comparisons between two groups **(E–H)** were performed using two-tailed unpaired Student’s t-tests. ns, not significant; *P < 0.05; **P < 0.01; ***P < 0.001.

The next, we measured the changes in lipid content and fatty acid levels in EESCs. The results showed that BCAT1 knockdown reduced lipid content and fatty acid levels in EESCs, whereas BCAT1 overexpression markedly elevated both parameters (Figures 3E,F). These findings suggested that knocking down BCAT1 reduced lipid accumulation in EESCs. Finally, we also assessed the changes in total cholesterol and triglyceride levels in EESCs using the kits. Interestingly, knocking down BCAT1 resulted in a significant reduction of total cholesterol and triglyceride levels in EESCs, while overexpressing BCAT1 significantly increased these levels (Figures 3G,H). In conclusion, our findings indicated that knocking down BCAT1 reduced lipid accumulation in EM.

### 3.4 BCAT1 promotes the expression of ACLY in EM

Our transcriptomic sequencing results show that knocking down BCAT1 significantly downregulates ACLY in EESCs. To further elucidate the relationship between BCAT1 and ACLY, we assessed the ACLY expression in EESCs using qRT-PCR and Western blot analysis. Notably, following BCAT1 knockdown, the ACLY expression in EESCs was significantly reduced. Conversely, overexpressing BCAT1 led to a significant upregulation of ACLY in EESCs (Figures 4A,B). Interestingly, qRT-PCR revealed significantly increased ACLY levels in EM tissues (Figure 4C), matching the high BCAT1 expression observed in EM lesions. Subsequently, we conducted a correlation analysis, which indicated a positive correlation between BCAT1 and ACLY (Figure 4D). To further validate these results, we also evaluated the ACLY expression in EESCs. As expected, ACLY was highly expressed in EESCs (Figure 4E). In summary, our findings indicated that BCAT1 promotes the expression of ACLY.

**FIGURE 4:**
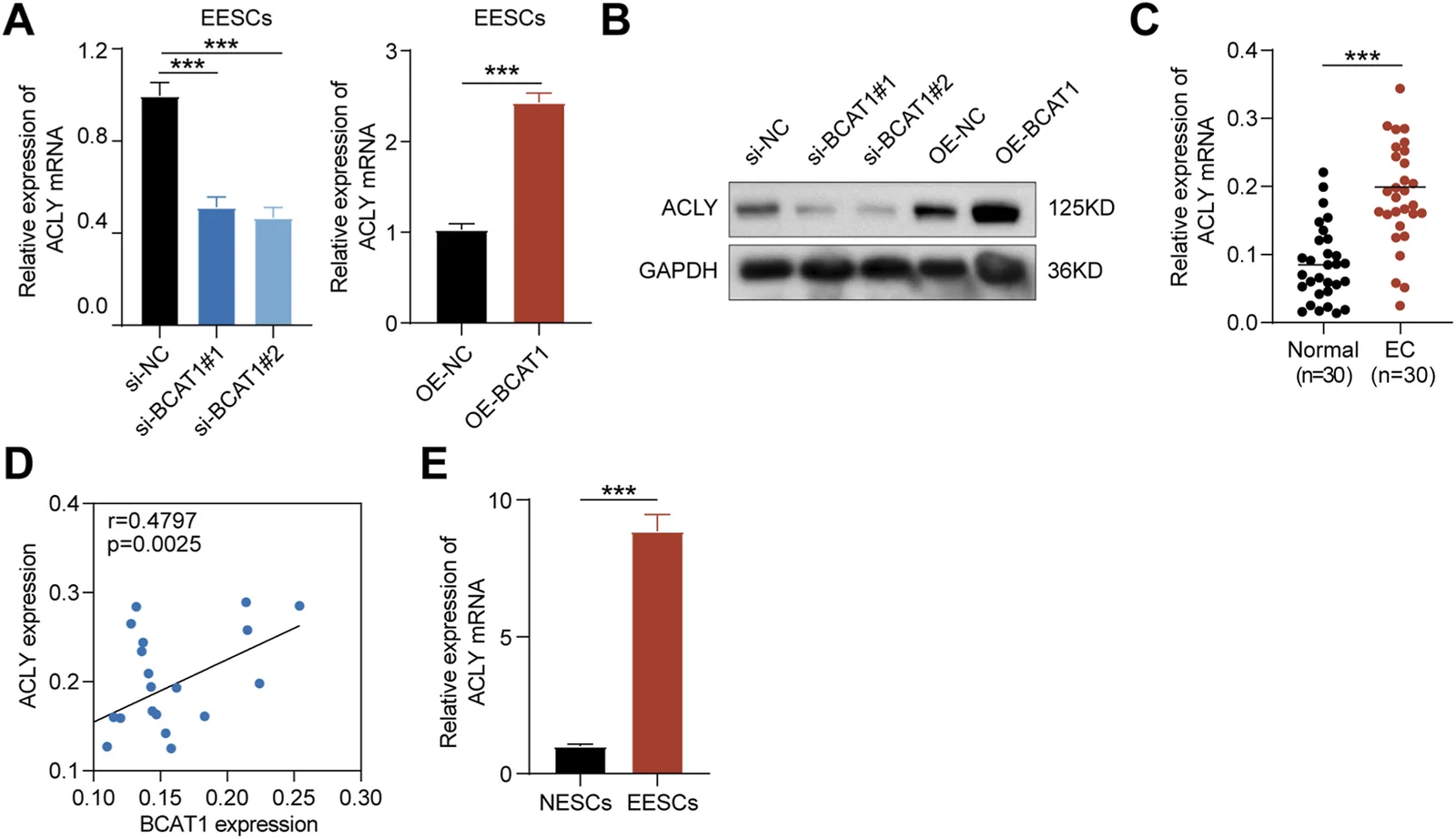
BCAT1 promotes the expression of ACLY in EM. **(A)** qRT-PCR analysis showed that BCAT1 knockdown decreased and BCAT1 overexpression increased ACLY mRNA in EESCs; n = 3 biological replicates with three technical PCR replicates per sample. **(B)** Western blotting showed corresponding changes in ACLY protein; n = 3 biological replicates with band intensities quantified by densitometry (representative blots shown). **(C)** qRT-PCR analysis showed that ACLY was significantly overexpressed in ectopic tissues versus normal tissues; each group comprised n = 30 independent patient tissue samples (biological replicates), and each sample was analysed in three technical PCR replicates and the results were averaged. **(D)** Correlation analysis revealed a positive correlation between BCAT1 and ACLY expression in the 30 ectopic tissues (n = 30 independent biological samples); the Pearson correlation coefficient (r) and two-tailed P value are given in the panel. **(E)** qRT-PCR analysis showed that ACLY was more highly expressed in EESCs than in NESCs; n = 3 biological replicates with three technical PCR replicates per sample. Data are presented as mean ± SD. Comparisons between two groups **(A–C,E)** were performed using two-tailed unpaired Student’s t-tests; correlation between BCAT1 and ACLY expression **(D)** was analysed using Pearson correlation. ns, not significant; *P < 0.05; **P < 0.01; ***P < 0.001.

### 3.5 BCAT1 regulates EESC cell proliferation via ACLY

SB-204990 (ACLYi) is a potent inhibitor of ACLY that primarily acts to inhibit cholesterol and fatty acid synthesis (Feng et al., 2020). To further clarify the relationship between BCAT1 and ACLY in EM, we conducted a series of rescue experiments using ACLYi. To identify the optimal concentration for ACLYi, we established the IC50 curve using the CCK-8 assay (Supplementary Figure S2A). Based on the analysis results from the IC50 curve, we confirmed that 10 μM should be used as the concentration of the ACLY inhibitor ACLYi. Furthermore, we transfected si-NC and si-ACLY into EESCs to knock down ACLY. qRT-PCR and Western blot analyses confirmed the significant reduction of ACLY expression upon ACLY knockdown (Supplementary Figure S3B).

Next, both CCK-8 and colony formation assays revealed that BCAT1 overexpression effectively promoted proliferation and colony formation in EESCs, whereas ACLY inhibition markedly attenuated the pro-proliferative effect of BCAT1 (Figures 5A,B; Supplementary Figure S2D). Furthermore, we also performed wound healing and Transwell assays on EESCs treated with ACLYi or with ACLY knockdown. Notably, we found that inhibiting ACLY significantly suppressed the ability of BCAT1 to promote migration and invasion of EESCs (Figures 5C,D; Supplementary Figures S2E,F). We have already confirmed the important role of BCAT1 in lipid metabolism. To further determine whether BCAT1 and ACLY work together in lipid metabolism, we conducted a series of lipid content-related assays on EESCs after treating them with ACLYi or with ACLY knockdown. Remarkably, overexpressing BCAT1 effectively stimulated lipid accumulation, cellular oxygenn consumption, and increased levels of cholesterol and triglycerides. However, inhibiting ACLY significantly weakened these effects (Figures 5E–H; Supplementary Figures S2G–J). In conclusion, our findings suggested that BCAT1 promotes EESC proliferation by upregulating ACLY expression and enhancing lipid accumulation.

**FIGURE 5:**
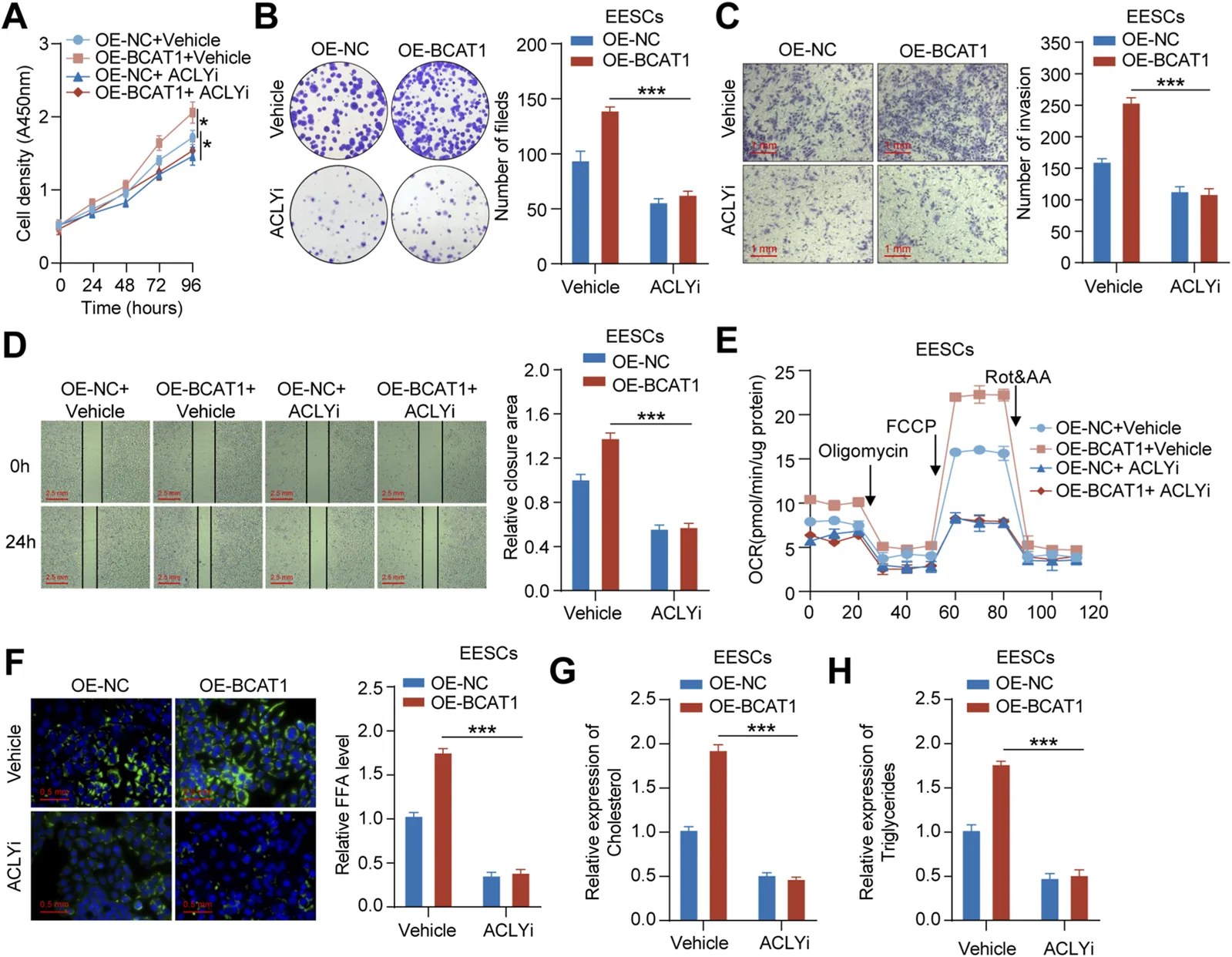
BCAT1 regulates EESC proliferation via ACLY. **(A,B)** CCK-8 **(A)** and colony formation **(B)** assays showed that the ACLY inhibitor (ACLYi) abolished the proliferative enhancement induced by BCAT1 overexpression; n = 3 biological replicates with three replicate wells per time point **(A)** and three replicate wells **(B)**. **(C,D)** Transwell **(C)** and wound healing **(D)** assays showed that ACLYi abolished the BCAT1-induced increases in invasion, migration, and wound closure; n = 3 biological replicates with three inserts and five random fields counted per insert (**(C)**; invasion with Matrigel, migration without Matrigel) and three replicate wells with multiple fixed measurement points per well **(D)**. **(E)** OCR measurements showed that ACLYi significantly inhibited the BCAT1-induced increase in OCR; n = 3 biological replicates with three technical wells. **(F)** BODIPY staining showed that ACLYi significantly inhibited the BCAT1-induced increase in lipid levels; n = 3 biological replicates with three technical repeats. **(G,H)** Total cholesterol **(G)** and triglyceride **(H)** assays showed that ACLYi significantly inhibited BCAT1-induced increases in both lipids; n = 3 biological replicates with three technical repeats per assay. Data are presented as mean ± SD. CCK-8 **(A)** and OCR **(E)** time-course data were analysed using two-way ANOVA followed by Sidak’s *post hoc* test; comparisons among multiple groups **(B–D,F–H)** were performed using one-way ANOVA followed by Tukey’s *post hoc* test. ns, not significant; *P < 0.05; **P < 0.01; ***P < 0.001.

### 3.6 BCAT1 regulates ACLY expression through histone acetylation

BCAT1 promotes the synthesis of acetyl-CoA, which in turn increases acetylation of histones, thus enhancing gene expression. To further investigate the role of histone acetylation in BCAT1 regulation of ACLY expression, we measured the levels of acetyl-CoA and acetylated histone H3 (Ac-H3) in EESCs. We found that knocking down BCAT1 resulted in a significant decrease in levels of acetyl-CoA and Ac-H3 in EESCs (Figures 6A,B). The next, while knocking down BCAT1 in EESCs, we treated them with Leucine (Leu). Notably, Leucine treatment significantly increased levels of acetyl-CoA and Ac-H3 within the cells. However, knocking down BCAT1 effectively reduced the increase induced by Leucine treatment (Figures 6C,D). Sodium acetate (AcNa), a compound formed from acetic acid and sodium, can be used to treat metabolic acidosis and regulate electrolyte balance (Neavyn et al., 2013). Previous studies have shown that the histone acetylation levels in nHL-60 cells significantly increase following treatment with AcNa (Yasuda et al., 2024). Subsequently, we treated BCAT1-knockdown EESCs with AcNa. The results showed that after knocking down BCAT1, the ACLY expression and levels of Ac-H3 in EESCs were reduced. Interestingly, treatment of BCAT1-knockdown EESCs with AcNa significantly enhanced the levels of ACLY and Ac-H3 (Figures 6E,F). Additionally, we used ChIP-qPCR to detect the acetylation levels in the ACLY promoter region. Importantly, we found that knocking down BCAT1 significantly decreased acetylation levels in the ACLY promoter region in EESCs (Figure 6G). C646 is a selective competitive inhibitor of p300/CBP histone acetyltransferase (HAT), which competes with acetyl-CoA to block the enzymatic activity of p300 and reduces histone acetylation levels (Oike et al., 2014). Subsequently, we treated BCAT1-overexpressing cells with 10 μM C646. The results revealed that C646 effectively blocked the upregulation of ACLY and H3K27ac induced by BCAT1 overexpression (Figure 6H). In summary, BCAT1 primarily regulates ACLY expression through histone acetylation.

**FIGURE 6:**
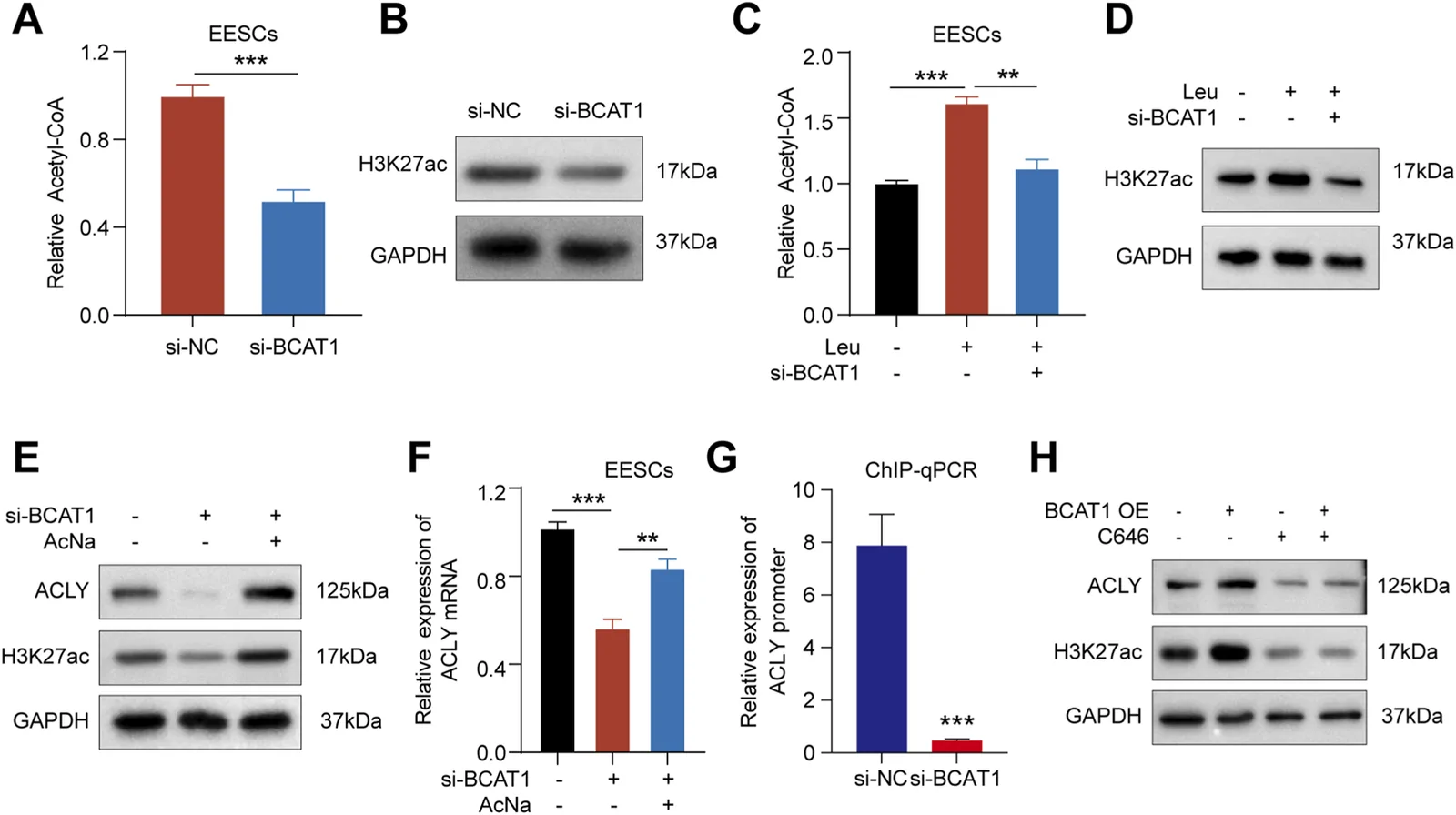
BCAT1 regulates ACLY expression through histone acetylation. **(A,C)** Acetyl-CoA assays showed that BCAT1 knockdown reduced acetyl-CoA levels **(A)** and prevented the increase induced by leucine **(C)**; n = 3 biological replicates with three technical repeats per sample. **(B,D)** Western blotting showed that BCAT1 knockdown reduced H3K27ac levels **(B)** and prevented the increase induced by leucine **(D)**; n = 3 biological replicates with band intensities quantified by densitometry (representative blots shown). **(E,F)** Western blot and qRT-PCR analyses showed that BCAT1 knockdown decreased H3K27ac and ACLY expression, whereas sodium acetate (AcNa) treatment restored H3K27ac and increased ACLY expression; n = 3 biological replicates, with densitometric quantification for Western blotting and three technical PCR replicates per sample for qRT-PCR (representative blots shown). **(G)** ChIP-qPCR showed that H3K27ac enrichment at the ACLY promoter decreased after BCAT1 knockdown; n = 3 biological replicates with three technical PCR replicates per sample, and ChIP signals were normalized to input and expressed as fold enrichment over the IgG control. **(H)** Western blotting showed that the p300/CBP inhibitor C646 (10 μM) abrogated the BCAT1 overexpression-induced upregulation of ACLY and H3K27ac; n = 3 biological replicates with band intensities quantified by densitometry (representative blots shown). Data are presented as mean ± SD. Comparisons between two groups **(A–D,G)** were performed using two-tailed unpaired Student’s t-tests; comparisons among three groups **(E,F,H)** were performed using one-way ANOVA followed by Tukey’s *post hoc* test. ns, not significant; *P < 0.05; **P < 0.01; ***P < 0.001.

## 4 Discussion

This study utilized a series of experiments to elucidate the relationship between BCAT1 and EM. The results showed that BCAT1 was highly expressed in EM tissues and cells. Following to knock down of BCAT1, the proliferation, migration, and invasion abilities of EESCs were significantly inhibited. Notably, we found that knocking down BCAT1 suppressed lipid metabolism in EESCs and decreased the ACLY expression level. Moreover, we confirmed that BCAT1 regulated ACLY expression mainly through an epigenetic mechanism involving histone acetylation. Overall, our study suggested that BCAT1 could be a potential therapeutic target for EM, providing new strategies for its treatment.

Previous researches have shown that BCAT1 is highly expressed in most cancer tissues and plays a significant role in the progression of various diseases (Hattori et al., 2017; Zheng et al., 2016; Ben-Yosef et al., 1998). For example, BCAT1 activates BCAA metabolism to promote myeloid leukemia progression (Hattori et al., 2017). BCAT1 can promote hepatocellular carcinoma cell proliferation and induces chemoresistance to cisplatin (Zheng et al., 2016). BCAT1 can inhibit cellular ferroptosis by activating the PI3K/AKT/mTOR signaling pathway and enhancing GPX4 expression, thereby alleviating early brain injury (Liu et al., 2024). Additionally, BCAT1 is highly expressed in nasopharyngeal carcinoma (NPC) tissues, and knocking down it significantly inhibits NPC cell proliferation (Zhou et al., 2007). However, EM is a benign gynecological disease, and the expression and function of BCAT1 have not been reported in EM. For the first time, we innovatively elucidated the critical function of BCAT1 in driving EM progression. In this study, we found that BCAT1 was also highly expressed in both EM tissues and cells. Furthermore, we validated that BCAT1 effectively promoted the proliferation and migration of EM cells *in vitro*.

Fatty acid metabolism reprogramming plays a significant role in cancer progression, generally by enhancing lipid synthesis, storage, and breakdown to promote tumor development (Hoy et al., 2021). Previous studies have shown that short-chain fatty acids derived from the gut microbiota can exert significant effects through G protein-coupled receptors (GPRs) and histone deacetylases. Moreover, they can also regulate RAP1GAP by inhibiting HDAC1, thereby preventing the EM progression (Chadchan et al., 2021). However, the molecular mechanisms of BCAT1 in lipid metabolism remain unclear. In this study, we found through transcriptomic sequencing that knocking down BCAT1 resulted in abnormal lipid metabolism in EESCs, with significant reductions in cellular lipid droplet content, OCR, cholesterol, and triglyceride levels. Additionally, KEGG pathway enrichment analysis indicated that pathways such as Neuroactive ligand-receptor interaction, Cytokine-Cytokine receptor interaction, and the cAMP signaling pathway were disrupted. In a study related to brain injury, the neuroactive ligand-receptor interaction pathway was identified as a core target for acetyl-tri-butyrate (ATBC)-induced neurotoxicity (Huang, 2023). However, this pathway has been rarely studied in EM, suggesting it could serve as a direction for future research on preventing and treating EM.

Typically, the expression of several enzymes involved in lipid metabolism is dysregulated in cancer cells (Turrado et al., 2012; DeBerardinis et al., 2007). ACLY expression is elevated in a variety of human tumor cells. ACLY expression is markedly elevated in renal cell carcinoma tissues and facilitates cancer cell proliferation and migration (Teng et al., 2018). Knockdown of ACLY induces lipid accumulation in non-small cell lung cancer cells and suppresses malignant progression of tumors (Migita et al., 2008). However, ACLY has rarely been investigated in the context of EM, and the regulatory mechanism underlying the BCAT1-ACLY axis remains uncharacterized. Notably, our results demonstrated that knocking down BCAT1 significantly downregulated ACLY expression. Conversely, overexpression of BCAT1 effectively promoted ACLY expression. ACLY converts citrate in the cytoplasm into acetyl-CoA, a key component for the biosynthesis of fatty acids and cholesterol. (Teng et al., 2018; Zaidi et al., 2012). Furthermore, we confirmed a positive correlation between BCAT1 and ACLY expression through Spearman correlation analysis. Interestingly, we also discovered that inhibiting ACLY significantly diminished the promoting effects of BCAT1 on EESCs. In summary, we verified that BCAT1 can effectively enhance lipid metabolism in EM cells and promote ACLY expression.

Previous studies have shown that the levels of histone acetylation are abnormal in EM patients (Xiaomeng et al., 2013). In addition, histone deacetylase (HDAC) inhibitors can downregulate G protein-coupled estrogen receptors and inhibit the proliferation of EM cells (Imesch et al., 2013). Therefore, we speculated that histone acetylation played a critical role in the progression of EM. However, the role of BCAT1 in epigenetics has been reported only infrequently. Notably, we found that knocking down BCAT1 significantly reduced the levels of acetyl-CoA and H3 histone acetylation in EM cells. This suggested that BCAT1 was highly significant in the epigenetics of EM. Furthermore, ACLY, a key enzyme in lipid synthesis and histone acetylation, is an important target for the treatment of complex vascular diseases (Grobs et al., 2024). We also confirmed through ChIP-qPCR that the acetylation level in the promoter region of ACLY in EM cells significantly decreased after knocking down BCAT1. This indicated that in EM, BCAT1 regulates the expression of ACLY through histone acetylation at the epigenetic level. In summary, our study firstly identifies the BCAT1-ACLY-H3K27ac signaling axis as a regulator of EM progression. Notably, we extend the well-established metabolic-epigenetic crosstalk observed in cancers to this benign yet invasive gynecological disease, which bears important implications.

Notably, this study has several notable limitations that warrant further investigation in future work. First, the clinical samples used in this work were obtained from a single-center retrospective cohort with 30 cases per group, and the ROC curve analysis in our study was exploratory. The single-center design with a limited sample size may introduce selection bias and statistical confounding. Accordingly, multicenter, large-scale independent clinical cohorts are required to externally validate our findings. Second, we only preliminarily demonstrated the influence of BCAT1 on lipid metabolism. Stable isotope metabolic tracing was not applied to quantify the rate of intracellular fatty acid synthesis, resulting in a lack of direct metabolic kinetic evidence. Third, intervention substrates such as sodium acetate and leucine exert broad systemic metabolic effects with insufficient specificity, making it difficult to attribute phenotypic changes exclusively to the target signaling cascade. Follow-up studies combining targeted metabolic tracing and genetic rescue assays are needed to further clarify the regulatory mechanism of the BCAT1-ACLY-H3K27ac signaling axis. Fourth, the ACLY-specific inhibitor SB-204990 used in this study carries potential off-target risks. We cannot fully rule out interference from non-specific effects in drug treatment experiments, which may compromise the precision of pathway validation. Fifth, all mechanistic experiments in the present study were performed using *in vitro* cell models, without supporting *in vivo* animal data. *In vitro* culture systems fail to faithfully recapitulate the complex hormonal regulation, immune microenvironment and inflammatory pathological conditions observed in endometriosis, and thus cannot accurately reflect the physiological function of the BCAT1-ACLY signaling axis *in vivo*. Therefore, future research should establish murine endometriosis models via allograft or xenotransplantation to verify the functional role of this signaling pathway and consolidate our mechanistic conclusions.

In conclusion, our study found that BCAT1 can promote lipid metabolism by upregulating the expression of ACLY, thereby enhancing the histone acetylation levels in EM and promoting the proliferation and migration of EESCs. Consequently, our findings emphasized that BCAT1 can serve as an important target for regulating lipid metabolism in EM cells, providing new strategies for the treatment of endometriosis. However, to further validate the potential of BCAT1/ACLY as potential therapeutic targets, it is crucial to conduct in - vivo experiments to confirm their roles and test the efficacy and safety of drugs targeting this pathway. This will help in developing more effective treatments for endometriosis.

## Data Availability

The raw data supporting the conclusions of this article will be made available by the authors, without undue reservation.
