# Supplementary material for: BCAT1 promotes acetylation of ACLY through fatty acid metabolism pathway promoting the occurrence and development of endometriosis
Source: Front Genet. 2026 Oct 20;17:1818692. doi: 10.3389/fgene.2026.1818692 (PMC13727071; doi:10.3389/fgene.2026.1818692)
Supplement: Supplementary file 1 [file Supplementaryfile1.docx]

**Supplementary Figures**

**Figure S1. BCAT1 overexpression restores the proliferative and migratory capacity of BCAT1-knockdown EESCs.** (A) Colony formation assay of EESCs co-transfected with si-NC + OE-NC, si-BCAT1 + OE-NC, or si-BCAT1 + OE-BCAT1; n = 3 biological replicates with three replicate wells. (B) Quantification of colony formation and CCK-8 growth curves under the same conditions as in (A); n = 3 biological replicates with three replicate wells per time point. (C) Transwell invasion assay of EESCs co-transfected as in (A); n = 3 biological replicates with three inserts and five random fields counted per insert (invasion with Matrigel). (D) Wound healing assay (0 h and 24 h) and quantification of relative closure area under the same conditions; n = 3 biological replicates with three replicate wells and multiple fixed measurement points per well. Data are presented as mean ± SD. Comparisons among three groups (A, C, D and the colony-formation quantification in B) were performed using one-way ANOVA followed by Tukey's post hoc test; CCK-8 time-course data (B) were analysed using two-way ANOVA followed by Sidak's post hoc test. ns, not significant; *P < 0.05; **P < 0.01; ***P < 0.001.

**Figure S2. ACLYi attenuates BCAT1-mediated promotion of proliferation, migration, and lipid accumulation in EESCs.** (A) IC50 curve of the ACLY inhibitor SB 204990 determined by CCK-8 assay in EESCs; n = 3 biological replicates with three replicate wells per drug concentration. (B) qRT-PCR and Western blotting validated ACLY knockdown efficiency with two independent siRNAs (si-ACLY-1 and si-ACLY-2); n = 3 biological replicates, with three technical PCR replicates per sample for qRT-PCR and densitometric quantification for Western blotting (representative blots shown). (C) Transwell invasion assay of EESCs after ACLY knockdown; n = 3 biological replicates with three inserts and five random fields counted per insert (invasion with Matrigel). (D) Colony formation assay of EESCs under four conditions: si-NC + OE-NC, si-NC + OE-BCAT1, si-ACLY + OE-NC, and si-ACLY + OE-BCAT1; n = 3 biological replicates with three replicate wells. (E) Transwell assay under the same four conditions as in (D); n = 3 biological replicates with three inserts and five random fields counted per insert (invasion with Matrigel, migration without Matrigel). (F) Wound healing assay (0 h and 24 h) and quantification of relative closure area under the same four conditions; n = 3 biological replicates with three replicate wells and multiple fixed measurement points per well. (G) Oxygen consumption rate (OCR) measured by Seahorse analyzer under the same four conditions as in (D); n = 3 biological replicates with three technical wells. (H) BODIPY staining and quantification of free fatty acid (FFA) levels under the same four conditions; n = 3 biological replicates with three technical repeats. (I, J) Quantification of total cholesterol (I) and triglyceride (J) levels under the same four conditions; n = 3 biological replicates with three technical repeats per assay. Data are presented as mean ± SD. The IC50 value (A) was determined by nonlinear regression analysis of the dose–response curve; comparisons between two groups (B, C) were performed using two-tailed unpaired Student's t-tests; comparisons among four groups (D–J) were performed using one-way ANOVA followed by Tukey's post hoc test. ns, not significant; *P < 0.05; **P < 0.01; ***P < 0.001.
